# Agreement between self-reports and statutory health insurance claims data on healthcare utilization in patients with mental disorders

**DOI:** 10.1186/s12913-023-10175-6

**Published:** 2023-11-11

**Authors:** Tarcyane Barata Garcia, Roman Kliemt, Franziska Claus, Anne Neumann, Bettina Soltmann, Fabian Baum, Julian Schwarz, Enno Swart, Jochen Schmitt, Andrea Pfennig, Dennis Häckl, Ines Weinhold

**Affiliations:** 1 WIG2 Institute for Health Economics and Health System Research, Markt 8, 04109 Leipzig, Germany; 2 Center of Evidence-Based Health Care, Medizinische Fakultät Carl Gustav Carus, Technische Universität Dresden, Dresden, Germany; 3 Department of Psychiatry and Psychotherapy, Universitätsklinikum Und Medizinische Fakultät Carl Gustav Carus, Technische Universität Dresden, Dresden, Germany; 4 Brandenburg Medical School, University Clinic for Psychiatry and Psychotherapy, Immanuel Hospital Rüdersdorf, Rüdersdorf, Germany; 5 Institute of Social Medicine and Health Systems Research, Medical Faculty, Otto-Von-Guericke- University Magdeburg, Magdeburg, Germany; 6 Institute of Public Finance and Public Management, Faculty of Economics and Management Science, Leipzig University, Leipzig, Germany

**Keywords:** Mental health, Self-report, Data linkage, Administrative data, Agreement

## Abstract

**Background:**

Data on resource use are frequently required for healthcare assessments. Studies on healthcare utilization (HCU) in individuals with mental disorders have analyzed both self-reports and administrative data. Source of data may affect the quality of analysis and compromise the accuracy of results. We sought to ascertain the degree of agreement between self-reports and statutory health insurance (SHI) fund claims data from patients with mental disorders.

**Methods:**

Claims data from six German SHI and self-reports were obtained along with a cost-effectiveness analysis performed as a part of a controlled prospective multicenter cohort study conducted in 18 psychiatric hospitals in Germany (PsychCare), including patients with pre-defined psychiatric disorders. Self-reports were collected using the German adaption of the Client Sociodemographic and Service Receipt Inventory (CSSRI) questionnaire with a 6-month recall period. Data linkage was performed using a unique pseudonymized identifier. Missing responses were coded as non-use for all analyses. HCU was calculated for inpatient and outpatient care, day-care services, home treatment, and pharmaceuticals. Concordance was measured using Cohen’s Kappa (κ) and intraclass correlation coefficient (ICC). Regression approaches were used to investigate the effect of independent variables on the agreements.

**Results:**

In total 274 participants (mean age 47.8 [SD = 14.2] years; 47.08% women) were included in the analysis. No significant differences were observed between the linked and unlinked patients in terms of baseline characteristics. Total agreements values were 63.9% (κ = 0.03; PABAK = 0.28) for outpatient contacts, 69.3% (κ = 0.25; PABAK = 0.39) for medication use, 81.0% (κ = 0.56; PABAK = 0.62) for inpatient days and 86.1% (κ = 0.67; PABAK = 0.72) for day-care services. There was varied quantitative agreement between data sources, with the poorest agreement for outpatient care (ICC [95% CI] = 0.22 [0.10–0.33]) and the best for psychiatric day-care services (ICC [95% CI] = 0.72 [0.66–0.78]). Marital status and time since first treatment positively affected the chance of agreement on utilization of outpatient services.

**Conclusions:**

Although there were high levels of absolute agreement, the measures of concordance between administrative records and self-reports were generally minimal to moderate. Healthcare investigations should consider using linked or at least different data sources to estimate HCU for specific utilization areas, where unbiased information can be expected.

**Trial registration:**

This study was part of the multi-center controlled PsychCare trial (German Clinical Trials Register No. DRKS00022535; Date of registration: 2020–10-02).

**Supplementary Information:**

The online version contains supplementary material available at 10.1186/s12913-023-10175-6.

## Introduction

Mental disorders are highly prevalent and have profound economic consequences [1, 2]. Understanding and assessing healthcare utilization (HCU) among individuals with mental disorders is essential for effective planning, resource allocation, optimizing care, ensuring equitable access, and addressing the economic burden [2, 3]. Healthcare resource use data is typically measured through patient self-reports or administrative records [3, 4]. Administrative data are primarily recorded within the healthcare system for reasons other than research purposes, such as billing and reimbursement. On the other hand, self-reported data generally include quantitative data used in large population-based studies collected via questionnaires or interviews [5, 6]. The strengths and limitations of using both data sources have already been extensively discussed [4, 5, 7].

A common measurement method for patient-reported resource use in mental health care are standardized questionnaires [8, 9], such as the Client Service Receipt Inventory (CSRI) [10]. The CSRI, along with its European version, the Client Socio-Demographic and Service Receipt Inventory (CSSRI-EU), has undergone extensive validation and adaptation in numerous languages, demonstrating its broad applicability across diverse research topics [8]. Different studies have investigated the concordance between CRSI/CSSRI and administrative data in the context of mental disorders. However, it is worth noting that, some of these studies are not representative of a broader psychiatric population, including only participants with a specific mental disorder [11], those insured by one individual statutory health insurance (SHI) fund [12], or only those attending hospitals in one particular district [13, 14], which may limit the generalizability of findings.

Heinrich et al. (2011) [14] conducted the first known study examining the agreement between self-reported data based on CSSRI and administrative data on healthcare service utilization in Germany. However, their study was based solely on hospital records, limiting the analysis to inpatient and day-care service utilization. Additionally, self-reports were collected through interviewer-administered instruments (telephone interviews), potentially introducing social desirability bias compared to self-administered approaches. To the best of our knowledge, apart from concordance on costs [12], no study has investigated the agreement between self-reports based on the German version of the CSSRI and health insurance claims data. Such an analysis would encompass additional service categories (e.g., outpatient care and medication use) and contribute to a more comprehensive understanding of the agreement between self-reported and administrative data.

To address these gaps and limitations, the present study aims to compare self-reported and administrative data on resource utilization in a mental health population, analyzing agreements across different healthcare sectors. Using the self-completed German version of the CSSRI questionnaire and health insurance claims data, the study examines agreement levels for dichotomous resource utilization, assesses concordance for volume utilization measures, and evaluates factors associated with agreement between self-reported and administrative data.

## Methods

### Study population

Data intended for performing a cost-effectiveness analysis were collected as part of the PsychCare study (PsychCare, German Clinical Trials Register No. DRKS00022535). The PyschCare study was a controlled prospective multicenter cohort study and collected data from 10 model hospitals offering flexible and integrative psychiatric treatment according to §64b German Social Code V (FIT hospitals), and eight control hospitals offering psychiatric treatment as usual. Financing of FIT hospitals is based on a global treatment budget (GTB) covering costs for all psychiatric hospital services and is related to the number of patients treated [15, 16]. Patients with particular mental disorders (i.e., mental and behavioral disorders due to use of alcohol [ICD-10 F10], schizophrenia, schizotypal disorder, delusional disorders or brief psychotic disorders [ICD-10 F20-23], or mood affective disorders [ICD-10 F30-39]) being treated in one of the participating institutions, who were also insured by one of six German health insurance funds (SHI) were included in the study. Patients with severe intellectual disabilities, acute suicidality, and severe organic brain dysfunction including impairment of cognitive function were excluded. Full details of the multicenter study, including ethics approval and consent to participate in the health surveys, are available in the study protocol [17].

### Study design and data sources

In the PsychCare study, self-reported retrospective data from the 6-month period prior to initial data collection, which lasted from March 2018 to September 2019, was requested via questionnaires, and with this a baseline for the trial participants established. Medication consumption was recalled from the last 1-month period of these 6 months. Healthcare claims insurance data were then obtained from 6 German SHI funds that insure the patients participating in the study, to check agreement of questionnaire collected HCU data. SHI data covered the period of 2016 – 2019, allowing for a pre-baseline period of 2 years. Data linkage of self-reported and claims data was performed using a unique pseudonymized individual-level identification key. Patients’ individual health insurance numbers were collected by an independent trust center, which sorted the numbers and requested the corresponding data from the participating health insurance funds. Pseudonymized data were transferred to the research unit and linked with primary data by study identification number. The procedure is in line with Good Practice in Secondary Data Analysis and reporting [18, 19] as well as Good Practice Data Linkage [20] and was permitted by the regulatory authorities of the six SHIs. Additionally, written informed consent for using health insurance claims data and linking it with primary data was obtained from patients.

### Measures of healthcare utilization

Self-reported HCU was assessed with a tailored German adaption of CSSRI [21, 22] including health and social care services (see Supplementary Table 1 in Additional File 1). To better represent FIT hospital care, the authors included additional services and greater differentiation. We compared the self-reported HCU data with those recorded in administrative data, including inpatient care, day-care, outpatient services, home-treatment, and pharmaceuticals. In the inpatient sector, we recorded the duration of hospital stays in days, including stays in general hospitals, psychiatric and psychosomatic hospitals, psychotherapy units, and addiction and substance misuse units. For day-care facilities, we categorized resource use based on the number of days spent in psychiatric or non-psychiatric day-care. In the outpatient sector, psychiatrist/neurologist visits, psychologist/psychotherapist visits (including both outpatient practitioners and psychiatric outpatient departments (PIA)), general practitioners (GPs) and other medical specialists were considered. Medication names were coded according to the Anatomical Therapeutic Chemical (ATC) classification system and grouped in categories according to their therapeutic class (psychotropic drugs and non-psychotropic drugs). Use of outpatient services and home treatment were assessed either as the exact number of contacts or as a frequency (Supplementary Fig. 1). The questionnaire data underwent quality and plausibility checks. Inconsistent or illogical answers were corrected whenever possible, while responses that could not be reasonably transferred were deleted and coded as missing. Structure and content of claims data has been described in detail previously [23]. Multiple claims made to the same healthcare professional within the same day were counted as a single contact.

### Missing data

In line with previous reports [24, 25], item-level missing values were coded as “no” in dichotomous reporting of healthcare utilization. Similarly, zero-imputation was employed for quantitative agreements and regression analyses. To mitigate potential bias introduction, we conducted an additional analysis by excluding missing data (see Supplementary Tables 2 and 3).

### Data analysis

All analyses were carried out using Microsoft SQL Server 2012 and R Software version 3.2.3 [26]. Calculation methods for lengths of stay and contact frequencies from patient data, taking into account plausibility criteria (e.g., maximum number of weekdays per year), is documented in Additional File 1. To measure resource utilization, variables from the two data sources were dichotomized (yes/no) indicating any service utilization in the past 6 months (e.g., outpatient visit or stay in hospital) or use of medication in the previous month. Next, the level of concordance was calculated using the sum of the proportions of absolute agreement (n, %), where both the self-reported and the administrative data indicated the same result (i.e., both indicated the occurrence of an event in the same period, or both indicated no event). Beyond chance agreement was demonstrated using Cohen’s kappa statistic (κ) computed using the *confusionMatrix()* function from the R caret package [27]. The magnitude of the frequently used kappa statistic is greatly affected by the prevalence of a condition in the population and by bias (i.e., the extent to which there is difference in the proportion of positive or negative cases between data sources), which has been widely criticized by researchers [28]; low kappa values, thus, do not necessarily reflect low proportions of overall agreement. To address this potential bias, the prevalence-adjusted bias-adjusted kappa (PABAK) was reported [29], which informs the rates of agreement regardless of an unbalanced proportion of positive or negative cases. The interpretation guidelines proposed by McHugh (2012) [30] were employed to assess the strength of agreement using Cohen’s kappa (≤ 0.20 none, 0.21–0.39 minimal, 0.40–0.59 weak, 0.60–0.79 moderate, 0.80–0.90 strong, and ≥ 0.91 almost perfect). Sensitivity (true positive rate) and specificity (true negative rate) were also calculated for each setting and medication use using the *diagnostic()* function from the R ThresholdROC package [31]. For calculation purposes, administrative records were treated as the reference for resource utilization.

For the volume measure of resource utilization (i.e., length of stay in hospital, number of outpatient visits, and home treatment contacts), the number of self-reported events were subtracted from the number of events in claims record to obtain the concordance between the two data sources. When the result was “0”, “total agreement” was assigned to the patient-reported information. When the result was negative, the information provided by the participant was considered an overestimation of utilization, because the number of self-reported events was higher than their corresponding administrative claims. When the result was positive, an underestimation was assumed. To estimate the quantitative concordance between self-reported and administrative data, intraclass correlation coefficients (ICC) were calculated using the *icc()* function from the R irr package [32]. To assess the strength of agreement using ICC, we utilized the interpretation guidelines proposed by Koo and Li (2016) [33] (≤ 0.49 poor, 0.50–0.74 fair, 0.75–0.90 good, ≥ 0.91 excellent). To provide a visual assessment of differences between self-reported events and those recorded in administrative data Bland–Altman plots were used. The correlation between the number of utilizations from the two data sources was assessed using the Spearman correlation coefficient (ρ), computed by the *cor.test()* function from the R stats package [26].

In addition to the analyses of HCU categories, which combine several single services, the divergences between the data sources were investigated for the subcategories within inpatient and outpatient settings, and subclasses of medications. Additionally, healthcare services were grouped into two broad subgroups: psychiatric and somatic services. Finally, to analyze the effect of distinct variables on differences in the concordance of resource use between the two data sources, univariate and multivariate logistic regression models reporting odds ratios and 95% confidence intervals were specified. Each dependent variable was coded binary (1 indicating agreement; 0 indicating disagreement). Linear regression models were used to assess factors associated with overreporting or underreporting the number of events (days/contacts). Here, the dependent variable was the difference between self-reported and administrative data. Positive values indicated an overreporting of utilization in self-reports compared to administrative data, while negative values indicated an underreporting. For all models, several characteristics that have been suggested to affect the accuracy of self-reports or administrative data were evaluated [34], including age, sex, living situation, education status, the length of stay in hospitals or day-care facilities and the number of outpatient visits. Significance was set at a value of *p* ≤ 0.05.

## Results

### Baseline characteristics

In total, 1150 patients who met inclusion criteria were eligible for the analysis. Of these, 274 (23.8%) individuals with valid informed consent to using claims data for scientific purposes and medically insured by one of the cooperating SHIs were successfully linked to administrative records and therefore included in the current study. The remaining 876 patients from the primary dataset were unable to be linked due to the unavailability of their claims data. Table 1 presents the descriptive statistics of participants in both datasets. Importantly, no apparent differences were observed between the linked and unlinked patients.

**Table 1:** Sociodemographic and clinical characteristic of participants

Variables	Linked (*n* = 274)	Not Linked (*n* = 876)	*p*-value
**Sex—n (%)**			0.18
Male	145 (52.9)	420 (48.0)	
Female	129 (47.1)	454 (51.8)	
Missing	0 (0.0)	2 (0.23)	
**Age in years—mean (SD)**	47.8 (14.2)	46.1 (14.9)	0.08
**Age in years—median [Min–Max]**	49 [19–85]	47 [18–92]	
**Age groups—n (%)**			0.14
18-24	12 (4.4)	66 (7.5)	
25-44	96 (35.0)	335 (38.2)	
45-64	140 (51.1)	382 (43.6)	
65 years +	26 (9.5)	91 (10.4)	
Missing	0 (0.0)	2 (0.2)	
**Index year – n (%)**^a^			1.00
2018	138 (50.4)	441 (50.3)	
2019	136 (49.6)	435 (49.7)	
**Years since first psychiatric treatment—n (%)**			0.51
≤ 5 years	90 (32.8)	309 (35.27)	
> 5 years	184 (67.2)	567 (64.73)	
**Main diagnosis—n (%)**			0.97
F10	59 (21.5)	180 (20.6)	
F20-23	50 (18.2)	132 (15.0)	
F30-39	165 (60.2)	477 (54.4)	
Other	0 (0.0)	87 (9.9)	
**Marital status – n (%)**			0.37
Single	126 (46.0)	429 (49.0)	
Married	64 (23.4)	210 (24.0)	
Married, but living separated	10 (3.6)	47 (5.4)	
Divorced	53 (19.3)	137 (15.6)	
Widowed	12 (4.4)	28 (3.2)	
Missing	9 (3.3)	25 (2.8)	
**Education Level—n (%)**^b^			0.40
Primary	65 (23.7)	213 (24.3)	
Secondary	160 (58.4)	471 (53.8)	
Tertiary	34 (12.4)	143 (16.3)	
Missing	15 (5.5)	49 (5.6)	
**Living situation—n (%)**			0.14
Independent accommodation	238 (86.9)	797 (91.0)	
Supervised accommodation	16 (5.9)	27 (3.1)	
Homeless	6 (2.2)	18 (2.0)	
Other	6 (2.2)	12 (1.4)	
Missing	8 (2.9)	22 (2.5)	

F10, mental and behavioral disorders due to use of alcohol; F20-F23, Schizophrenia, schizotypal and delusional disorders; F30-F39, mood affective disorders

^a^Year of baseline assessment

^b^CASMIN classification: Primary (1a), Secondary (1b, 1c, 2a, 2b, 2c_gen, 2c_voc) and Tertiary (3a, 3b)

Differences between groups were tested with a chi-squared test for all binary parameters and with a Mann–Whitney U test for the continuous parameter

The average age of linked participants was 47.8 years (SD ± 14.2, range = 19–85, median = 49 years), approximately half of them were male (52.92%), and 67.15% were in psychiatric treatment for more than 5 years. In total 21.53% were identified with mental and behavioral disorders due to use of alcohol, 18.25% suffered from schizophrenia, schizotypal and delusional disorders, and 60.22% were diagnosed with mood affective disorders. Participants were mostly single (45.99%) and lived predominantly in an independent accommodation (86.86%).

### Agreement between self-reported and administrative data for dichotomous reporting on healthcare utilization

Table 2 shows a detailed comparison of self-report and administrative measures for healthcare utilization (yes/no) within specific sectors. The table presents the utilization of healthcare services over a 6-month period before the baseline assessment, and the use of medication in the previous month. See Supplementary Fig. 2, for further details on data sources overlap.

**Table 2:** Proportion of patients by source and concordance between self-reported and administrative data for utilization of health care services and use of medications (*n* = 274)

Source	Inpatient	Day-care	Outpatient	Medications
Missing SR—n (%)^a^	90 (32.8)	189 (69.0)	97 (35.4)	83 (30.3)
Total utilization—n (%)^b^	215 (78.5)	100 (36.5)	268 (97.8)	238 (86.9)
Utilization in SR—n (%)	183 (66.8)	85 (31.0)	176 (64.2)	188 (68.6)
Utilization in AR—n (%)	195 (71.2)	77 (28.1)	261 (95.3)	204 (74.4)
Utilization in AR and in SR—n (%)	163 (59.5)	62 (22.6)	169 (61.7)	154 (56.2)
Agreement—n (%)^c^	222 (81.0)	236 (86.1)	175 (63.9)	190 (69.3)
Kappa [95% CI]	0.56 [0.45–0.66]	0.67 [0.57–0.76]	0.03 [-0.04–0.1]	0.25 [0.13–0.37]
PABAK [95% CI]	0.62 [0.52–0.71]	0.72 [0.63–0.80]	0.28 [0.16–0.39]	0.39 [0.27–0.50]
Sensitivity—% [95% CI]	83.6 [77.5–88.3]	80.5 [69.6–88.3]	64.8 [58.6–70.5]	75.5 [68.9–81.1]
Specificity—% [95% CI]	74.7 [63.4–83.5]	88.3 [82.8–92.3]	46.2 [20.4–73.9]	51.4 [39.3–63.4]

*Abbreviations*: *AR* Administrative records, *SR* Self-reported data, *CI* Confidence Interval, *kappa* Cohen’s kappa measure of inter-rater agreement, *PABAK* Prevalence and Bias Adjusted Kappa

^a^In the statistical analyses, answers “zero (0)” and missing in the questionnaire were merged into “no utilization”

^b^Total number of participants using medical services who were identified by self-reported and/or administrative data

^c^The percentage of agreement indicates concordance between self-reported and administrative data based on the same result (both indicated an event, or both indicated no event)

The prevalence of utilization based on administrative records was higher than that based on self-reported data, except for day-care services. There was a high degree of concordance between data sources, with values ranging from approximately 86.1% for day-care to 63.9% for outpatient care (Table 2). Kappa-values varied across settings, ranging from 0.03 for overall outpatient services to 0.67 for use of day-care services. After considering prevalence and bias, PABAK ranged from 0.28 (outpatient) to 0.72 (day-care) and was markedly higher than the unadjusted kappa values for most of the resource categories. Self-reported use of inpatient and outpatient services, and use of medications had higher levels of sensitivity than specificity, whereas self-reported day-care service had higher specificity than sensitivity. Agreements for home treatment and concordance between self-reported medication use and prescribing data in administrative records across different drug classes are provided in Supplementary Tables 4 and 5, respectively (Additional File 1).

Excluding all cases with any missing self-report utilization (Supplementary Table 2) led to somewhat higher levels of raw agreement. However, calculating kappa values became notably challenging or impossible due to the limited number of instances where patients self-reported “0 (zero)” for utilization, resulting in smaller coefficients.

### Agreement between self-reported healthcare utilization and administrative data for quantity reporting

Table 3 shows the accuracy of self-reports in terms of the resource utilization volume, i.e., length of stay in hospital in days and number of outpatient contacts.

**Table 3:** Differences in healthcare resource use between administrative records and self-reported data for different medical services from the inpatient, day-care, and outpatient settings (*n* = 274)

Settings	Psychiatric care	Somatic Services	All-cause
***Inpatient***			
Missing SR—n (%)^a^	100 (36.5)	256 (93.4)	94 (34.3)
Number of days SR- Mean (SD)	32.2 (43.1)	2.0 (7.1)	33.2 (43.6)
Number of days AR—Mean (SD)	36.3 (38.2)	2.0 (7.1)	38.2 (39.5)
ICC [95% CI]	0.51 [0.42–0.59]	0.42 [0.32–0.52]	0.51 [0.41–0.59]
***Day-care***^b^			
Missing SR—n (%)^a^	195 (71.2)	269 (98.2)	192 (70.1)
Number of days SR- Mean (SD)	13.6 (29.4)	1.4 (11.9)	14.93 (31.3)
Number of days AR—Mean (SD)	10.5 (23.2)	0.0 (0.0)	10.53 (23.2)
ICC [95% CI]	0.72 [0.66–0.78]	*	0.65 [0.58–0.72]
***Outpatient***			
Missing SR—n (%)^a^	133 (48.5)	150 (54.7)	101 (36.9)
Number of contacts SR- Mean (SD)	4.5 (8.1)	2.6 (4.4)	7.1 (9.9)
Number of contacts AR—Mean (SD)	4.6 (10.1)	7.3 (6.6)	12.0 (12.1)
ICC [95% CI]	0.22 [0.1–0.33]	0.23 [0.03–0.4]	0.22 [0.1–0.33]

For calculation purposes, administrative records were treated as the reference for resource utilization

*Abbreviations*: *AR* Administrative records, *SR* Self-reported data, *ICC* Intraclass correlation coefficient, *95% CI* 95% confidence interval, *SD* Standard Deviation

^a^In the statistical analyses, answers “zero (0)” and missing in the questionnaire were merged into “no utilization”

^b^Somatic services were not found in administrative data

^*^Too few cases to analyze

**Fig. 1:**
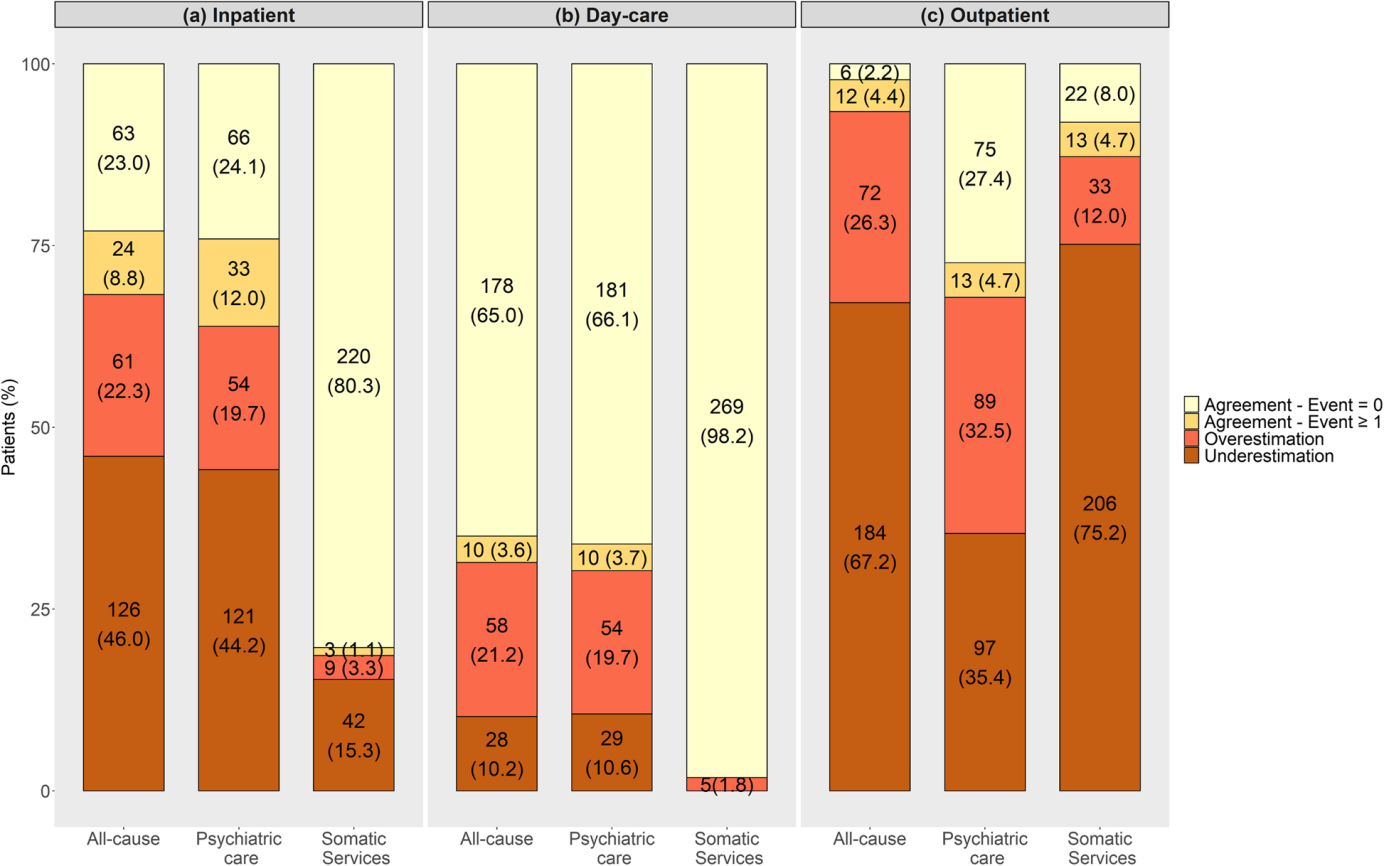
Concordance on healthcare resource use between administrative records and self-reported data in the inpatient, day-care, and outpatient settings, stratified by psychiatric, somatic, and all-cause services. Note that agreement on the number of events (days in hospital/contacts with healthcare professionals) included zero responses (i.e., self-reported data and administrative data indicate no event)

Overall, participants self-reported on average 33.2 (SD ± 43.6) inpatient days, 14.9 (SD ± 31.2) days in day-care hospitals, and 7.1 (SD ± 9.9) outpatient visits, while the administrative claims data indicated on average 38.2 (SD ± 39.5) inpatient days, 10.5 (SD ± 23.3) days in day-care hospitals, and 12.0 (SD ± 12.1) outpatient visits. When considering administrative records as a reference, most participants accurately estimated the length of stay in day-care hospitals (68.6% including responses of zero) but tended to predominantly under-report both inpatient days (46.0%) and outpatients visits (67.2%) (Fig. 1). Despite the significant proportion of discordance in the outpatient sector, it is noteworthy that approximately 45.7% of participants who over or underreported the number of medical visits were found to deviate by a margin of ± 1 to 5 contacts (Supplementary Fig. 3).

Excluding all cases with missing quantity self-report utilization (Supplementary Table 3) resulted in noticeably lower levels of raw agreement, yet the coefficients of agreement remained similar or approximated those obtained when coding missing data as non-utilization.

**Fig. 2:**
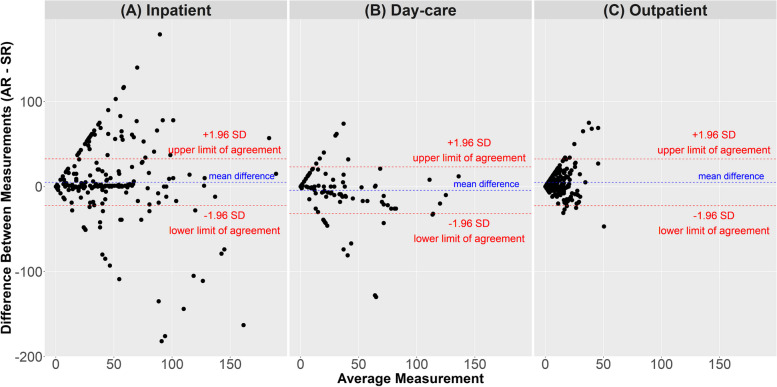
Bland–Altman Plots for the number of days (**a**, **b**) or number of contacts (**c**) found in both data sources. The average of the two measurements is plotted along the horizontal axis and the difference between the two methods is plotted along the vertical axis

Figure 2 displays Bland–Altman plots comparing the number of events (hospital days/outpatient visits) by self-reported data with the number of events in administrative records.

The frequency of higher positive average differences between data sources suggests a bias towards under-reporting the true number of inpatient days and outpatient contacts. The correlation between administrative and self-reported data is illustrated in Supplementary Fig. 4, and the corresponding correlation coefficients are provided in Supplementary Table 6. Agreement between data sources ranged from minimal for outpatient contacts (ICC = 0.22) to moderate for day-care services (ICC = 0.65). When psychiatric and somatic service utilization were examined separately, ICC was only slightly better for inpatient psychiatric care (psychiatric departments = 0.57 vs somatic services = 0.42), and outpatient somatic care (somatic services = 0.23 vs psychiatric care = 0.22). Quantitative agreement between data sources for the different components of inpatient and outpatient services are shown in Supplementary Tables 7 and 8 (see Additional File 1), respectively. Due to the small number of patients reporting on the number of contacts with healthcare professional in home treatment (less than 10%) and use of somatic services in day-care hospitals (less than 2%), ICCs of these categories could not be calculated (see Supplementary Table 4 in Additional File 1 and Table 3, respectively).

### Influence of variables on difference in agreement of healthcare utilization

Logistic regression results for the agreement of resource utilization (concordance on resource use by setting: no = 0/yes = 1) are shown in Table 4.

**Table 4:** Odds ratio and corresponding 95% confidence intervals from the logistic regression models for the overall agreement on any utilization (yes/no) by healthcare resource

Predictor	Inpatient	Day-care	Outpatient	Medication
***Univariate models***				
Sex Male^a^	0.87[0.47–1.59]	1.01[0.51–2.01]	0.66[0.4–1.08]	1.18[0.71–1.98]
Age (years)	1.01[0.99–1.03]	1.01[0.98–1.03]	1.01[0.99–1.03]	1.01[0.99–1.03]
Age group 65 + years	0.75[0.35–1.58]	0.93[0.38–2.26]	1.16[0.6–2.24]	0.69[0.36–1.32]
Time since 1^st^ treatment^b^ > 5 years	0.99[0.52–1.89]	0.93[0.45–1.95]	1.47[0.88–2.48]	1.3[0.76–2.23]
Marital Status^c^ Married/living as married	**2.48[1.00–6.14]**	0.87[0.4–1.91]	**2.81[1.41–5.59]**	0.85[0.47–1.56]
Education Level^d^ Tertiary	3.89[0.9–16.85]	1.71[0.49–5.93]	1.32[0.6–2.91]	1.41[0.61–3.27]
Living situation^e^ Independent accommodation	1.98[0.82–4.81]	1[0.33–3.06]	2.01[0.91–4.43]	0.36[0.12–1.06]
***Multivariate models***				
Sex Male^a^	0.9[0.44–1.82]	0.96[0.45–2.07]	0.7[0.39–1.24]	1.05[0.59–1.89]
Age (years)	1.01[0.98–1.05]	1.02[0.98–1.06]	1[0.98–1.03]	1.02[0.99–1.05]
Age group 65 + years	0.37[0.11–1.21]	0.59[0.16–2.16]	0.65[0.25–1.72]	0.39[0.15–1.03]
Time since 1^st^ treatment^b^ > 5 years	1.21[0.59–2.46]	0.74[0.33–1.66]	**1.82[1.03–3.23]**	1.38[0.77–2.47]
Marital Status^c^ Married	1.99[0.75–5.25]	0.74[0.3–1.81]	**2.34[1.12–4.91]**	0.77[0.4–1.5]
Education Level^d^ Tertiary	3.2[0.72–14.26]	1.73[0.48–6.18]	1.13[0.49–2.6]	1.46[0.61–3.52]
Living situation^e^ Independent accommodation	1.62[0.61–4.33]	1.14[0.35–3.71]	1.86[0.79–4.39]	0.32[0.09–1.14]
***Nagelkerkes R***^***2***^	0.22	0.12	0.22	0.19

Significant associations (*P* ≤ 0.05) are denoted in bold

^a^female

^b^ ≤ 5 years

^c^Single/Married but living separated/ Divorced/ Widowed

^d^Primary/Secondary

^e^Supervised accommodation/Homeless/Other

In the univariate analyses, the only statistically significant associations found were between marital status and utilization of inpatient and outpatient services. Married or living as married adults were more likely to recall the occurrence of any inpatient event (OR [95%-CI] = 2.48[1.00–6.14]) and outpatient visit (OR [95%-CI] = 2.81[1.41–5.59]) compared with their counterparts who were either single, married but living separated, divorced, or widowed. In terms of the magnitude and direction of effect, the results of multivariate analysis are similar. Marital status again attained statistical significance for the association with outpatient services. Additionally, individuals in psychiatric treatment longer than 5 years were also more likely to correctly report any outpatient visit in the multivariate model (OR [95%-CI] = 1.82[1.03–3.23]). For inpatient and day-care services, and medication use, no predictor had a consistent association with the agreement between data sources.

Next, linear regression models were used to assess the predictors of under- or overreporting for volume utilization measures. Table 5 shows regression coefficients for socio-demographic predictors of the difference in the number of events (days/contacts) measured with administrative records versus self-reported data. For the outpatient sector, the statistically significant factors were sex, age, and number of events. Males tended to overreport outpatient visits, while increase in age resulted in underreporting of outpatient contacts. An increase in the number of inpatient days and outpatient contacts resulted in overreporting of events in the respective sectors.

**Table 5:** Multivariate linear regression models for predictors of differences between self-reported and administrative data for the volume of utilization (number of days/contacts) by healthcare resource

Predictor	Inpatient		Day-care		Outpatient	
	**Beta**	**SE**	**Beta**	**SE**	**Beta**	**SE**
Sex (0 = Female, 1 = Male)	-0.08	5.23	-1.79	3.15	**-4.88**	**1.17**
Age (years)	0.30	0.24	-0.05	0.15	**0.11**	**0.05**
Age group (0 = 18–64, 1 = 65 +)	-0.86	8.82	3.73	5.32	-1.37	1.98
Time since 1^st^ treatment (0 = ≤ 5 years, 1 = > 5 years)	3.14	5.24	-1.13	3.16	2.07	1.18
Marital Status (0 = Married/living as married, 1 = other)	-1.50	6.02	-1.95	3.63	-1.45	1.35
Education Level (0 = primary/secondary, 1 = tertiary)	7.08	7.35	-0.60	4.44	-0.71	1.65
Living situation (0 = independent accommodation, 1 = other)	-15.60	8.38	1.11	5.05	1.54	1.88
Number of inpatient days^a^	**-0.42**	**0.06**	-	-	-	-
Number of days in day-care^a^	-	-	-0.08	0.07	-	-
Number of outpatient contacts^a^	-	-	-	-	**-0.62**	**0.05**
**Explained variance—*R***^***2***^	0.2		0.01		0.47	

Significant associations (*P* ≤ 0.05) are denoted in bold

^a^Days/contacts from administrative records

## Discussion

### Inpatient and day-care services

Our findings regarding concordance on any stay in hospital or day-care facilities and their volume of utilization align partially with previous research [14, 35–38]. The agreements observed for inpatient and day-care services were weak and moderate respectively. The Kappa were slightly lower than that reported in studies using hospital computerized claims databases [14, 35], but higher than that using GP records [36, 37] or administrative data from medical service plans [38].

Quantitative comparison with previous investigations is challenging due to variations in study design and measures of concordance. These differences in methodology and comparability may explain the observed variations in results. For instance, some studies did not include non-psychiatric care, while others combined data from hospital admissions and day-care facilities into a single variable. Studies examining psychiatric and psychosomatic services, either combined or separately, revealed minimal to weak agreements between self-reported data and GP records for various types of hospital services [36, 38], while moderate agreement was found between CSSRI data and hospital records for combined psychiatric inpatient and day-care services [14]. In the current study, when considering admission reports from hospitals and day-care facilities separately and adjusting for prevalence and bias, moderate agreement coefficients were found between self-reports and health insurance claims data for resource utilization.

Regarding volume of service utilization, fair concordance was found for the overall inpatient sector, and again our correlation values were lower than those found in the study using hospital records [14] and higher or similar to those using GP records [37]. Agreement on the length of stay in day-care facilities was the strongest found in our analysis, contrary to previous investigations reporting low agreement for day-care services [13, 14]. While the agreement mainly stemmed from a significant proportion of respondents reporting no service utilization, 36.5% of patients received day-care treatment in at least one data source, potentially contributing to higher correlation values in our study. Notably, 59.8% of our sample received treatment in FIT hospitals, where inpatient treatment intensity was shown to be reduced in association with an increase in day-care [39]. The potential positive effect of this factor on patients’ recall of day-care services cannot be ruled out, necessitating further investigation.

### Outpatient services and medication use

In our study, only a minimal agreement between self-reports and claims-based data was found regarding the utilization of outpatient services and medication usage. Limited literature exists on the accuracy of self-reports compared to data records for combined outpatient services and prescribed medications in mental health populations [37, 40]. For the general population, kappa values for the concordance of outpatient events have typically ranged from weak to moderate depending on the medical specialty [41–43]. Medication utilization shows moderate to strong concordance, depending on the specific drug classes [44, 45].

Our results on the dichotomous agreement between data sources for outpatient services and medication use are similar to those found in individuals with chronic conditions [46–48]. As both outpatient service utilization and medication use were underreported when compared to administrative data, poor health status may have affected the recall of utilization and consequently the agreement for less significant or salient events. On the other hand, a previous study has found no association between disagreement and psychiatric diagnosis for the recall of outpatient events [49]. In addition, since we coded missing data as negative self-reports of resource utilization, it was not possible to differentiate between participants who intended to deny outpatient services or medication use and those with no motivation to record and report their data. Alternatively, the lower prevalence of self-reported medication use may be indeed due to inaccuracies in administrative data. For example, a considerable percentage of individuals prescribed antidepressants choose not to initiate or prematurely discontinue treatment, while still being registered in the database [50]. In addition, in the administrative records, any prescription within 3 months before baseline assessment was considered a positive event, while CSSRI focused specifically on medications for mental disorders.

Regarding volume of service utilization for outpatient contacts, only a poor agreement was found in our study for outpatient services combined using insurance claims as a comparator. Correlation and agreement were found by our study to be better for predominant care (i.e., psychiatric services) than somatic services, similarly to what has previously been reported for epilepsy patients [51]. The observed underestimation of the number of overall outpatient contacts (67.2%, -4.8 contacts) was attributed to reports on the number of GP visits. This finding is supported by one [37], but not by other previous investigations [11, 51, 52]. In some cases, underreporting of GP contacts may occur when patients who also have regular contacts in mental health facilities misremember whether the doctor visit was a general or a psychiatric contact.

The number of self-reported outpatient contacts was observed to have an important impact on the total agreement of the number of events. However, in contrast to previous investigations [34], patients in our study with a greater number of outpatient visits were more likely to overreport than underreport the number of outpatient contacts. On the other hand, there are also plausible explanations for this result; our sample consisted of participants with mental disorders who use many different types of health service, such as complementary and therapeutic care. It is possible that subjects confused these with outpatient visits, which were then consequently over-reported. In fact, similar findings on overreporting have been found for individuals with self-rated poor health status in general population-based studies [42, 53].

Consistent with previous findings [34, 49, 54] and as expected due to memory impairment, increasing age was a significant determinant of underreporting outpatient contacts. Concerning the agreement on the utilization (yes/no) of outpatient services, married status and being in psychiatric treatment for more than 5 years were associated with a greater likelihood of concordance. Being married can be associated with positive social support for a patient’s engagement in medical treatment [55] and possibly with better recall of the services used, at least in the outpatient setting. Likewise, people in a longer period of psychiatric treatment could be more concerned with their health and more engaged in their treatment providing a more correct estimation on the use of services. However, more research is needed to confirm these conjectures.

### Limitations

Due to the nature of the questions in CSSRI lacking response alternatives, missingness was assumed to indicate non-use. This approach may have led to potential misclassification, but we do not believe it influenced the disagreement between data sources. In the last section of the questionnaire, which included “yes/no” checkboxes, 96% of participants attempted to answer at least one subsequent item. Some missing data were likely due to structural design or lack of utilization, rather than refusals to respond. For example, dates of admission and discharge were missing for patients without hospitalization events. In such cases where missing responses were due to design or lack of utilization, it was reasonable to assume that the missing items represented non-use. To address potential biases, we conducted an additional analysis by excluding missing data. Consistent with a previous report [56], we found only minor differences in the coefficient of agreements when missing responses were excluded (Supplementary Table 3).

The assessment of outpatient services involved capturing the exact number of contacts or their frequency, which may introduce inaccuracies due to the exclusion of patients who provided unclear responses. However, our analysis revealed that approximately 73% of participants provided specific numerical values for their contacts, indicating a substantial proportion of precise responses (Supplementary Fig. 1).

The 6-month recall period used in our analyses may introduce potential bias, particularly for psychiatric patients who frequently utilize services. This longer recall period can affect the accuracy and reliability of self-reported data, potentially leading to underreporting or recall errors. While studies with shorter recall periods or more frequent assessments can provide more precise and reliable data on service utilization in psychiatric populations, it is important to acknowledge that research on the effects of new and alternative approaches to care requires longer observation periods. Implementing shorter observation periods would require more frequent data collection points in longitudinal studies, which should be carefully evaluated to minimize the risk of loss-to-follow-up, especially among vulnerable patient groups.

In terms of the coverage of claims data, it is important to note that certain complementary and non-medical services fall outside the scope of German SHI funds, limiting their assessment in this study. Furthermore, potential gaps in the administrative data may arise from coding errors, incomplete or delayed claim submissions. It is also essential to consider that self-reported data, reliant on individual recall and perception [34], may introduce the possibility of misinterpreting the specific type of healthcare service mentioned in the questionnaire.

The unadjusted kappa values remain consistently low, even when a large proportion of concordant pairs is observed, such as in the case of outpatient contacts and medication use. Furthermore, our data exhibited a high frequency of zero occurrences, posing challenges for calculating the ICC, which assumes a normal distribution of the data. It is important to consider that the comparability of kappa and ICC values across countries may be limited due to potential variations in healthcare systems. However, certain outcomes of interest, such as inpatient days and outpatient visits, are common in many countries. To enhance interpretability and facilitate future comparisons, our findings were presented using absolute numbers and cross tables for both data sources. When statistically evaluating HCU data from different sources, it is advisable to consider a combination of agreement indicators rather than relying on single measures alone.

Finally, it is important to highlight that the linkage to claims data was limited to only 274 patients due to administrative data availability. This smaller subset of participants may introduce selection bias, potentially limiting the generalizability of our findings. However, we compared the characteristics of linked and non-linked participants and found no significant differences at baseline. This suggests that the linked subgroup is representative of the larger cohort, mitigating concerns of bias introduced by limited sample size in the linkage subgroup. In addition, it is important to recognize that our study focused exclusively on psychiatric patients. While this allowed us to examine service utilization patterns within this specific population, it also limits the generalizability of our findings to other populations. Therefore, caution should be exercised when extrapolating the results to broader healthcare contexts and diverse patient groups.

## Conclusion

In summary, we found relatively high absolute concordance on resource utilization across all settings, but due to the differences among positive and negative agreements, the kappa values were generally low. Inclusion of PABAK, an indicator less sensitive to sampling bias and prevalence, resulted in consistently higher agreement rates in our study. Frequent events, such as outpatient appointments, were less accurately reported than less frequent and possibly more salient events, such as hospital admissions (inpatient and day-care). However, a substantial proportion of participants exhibited minimal disagreements, with discrepancies falling within a narrow margin of ± 1 to 5 contacts. Based on the results of our study, it can be inferred that the German CSSRI and SHI funds data demonstrate better compatibility and agreement for hospital admissions (inpatient and day-care) within a 6-month recall period. However, for outpatient visits and medication use, the level of agreement between these data sources is found to be less accurate. Results derived from investigations relying on just one of these data sources must be interpreted with caution. Alternatively, conducting individual-level linkages of primary and secondary data could improve data quality and strengthen the findings.

### Supplementary Information

**Additional file 1: Supplementary Fig. 1. **Self-reported utilization of outpatient services and home treatment. Responses were exact number of contacts or frequency. The analysis considered the duration information provided in the questionnaire when the precise number of contacts was stated, otherwise, frequency was taken into consideration. Abbreviations: EP, Established practitioners; PIA, psychiatric outpatient departments. **Supplementary Table 1. **Variables of primary and secondary data.** Supplementary Fig. 2. **Venn diagrams illustrate the overlap in the numbers of users (left panels) and non-users (right panels) of (A) inpatient, (B) day-care, (C) outpatient services, and (D) medications in two different data sources. Abbreviations: AR, administrative records; SR, self-reported data. **Supplementary Table 2. **Proportion of patients by source and concordance between self-reported and administrative data for utilization of health care services and use of medications. All cases with any missing self-report utilization data were excluded. **Supplementary Table 3. **Differences in healthcare resource use between administrative records and self-reported data for different medical services from the inpatient, day-care, and outpatient settings. All cases with any missing self-report utilization data were excluded. **Supplementary Table 4. **Differences in healthcare resource use between administrative records and self-reported data for home treatment (*n*=274). **Supplementary Table 5. **Differences in medication use between administrative records and self-reported data (*n*=274). **Supplementary Fig. 3. **Over- and underreporting on healthcare resource use between administrative records and self-reported data in the inpatient, day-care, and outpatient settings. Discordance was accounted for within a margin of error of ± 1-5 contacts, ± 6-10 contacts, and ± 11 or more contacts. **Supplementary Fig. 4. **Scatterplots for the correlations between data sources in the inpatient (a), day-care (b) and outpatient (c) settings for the same period. **Supplementary Table 6. **Correlation coefficients between administrative and self-reported data. **Supplementary Table 7. **Differences in healthcare resource use between administrative records and self-reported data for different medical services from the inpatient setting (*n*=274). **Supplementary Table 8. **Differences in healthcare resource use between administrative records and self-reported data for different medical services from the outpatient setting (*n*=274).

## Data Availability

The datasets generated and/or analyzed during the current study are not publicly available. The datasets used during the current study are available from the corresponding author on reasonable request.

## Abbreviations

ATC: Anatomical Therapeutic Chemical Classification System
CSRI: Client Service Receipt Inventory
CSSRI-EU: European version of the Client Socio-Demographic and Service Receipt Inventory
FIT: Flexible and integrative psychiatric treatment
GPs: General practitioners
GTB: Global treatment budget
SHI: Statutory health insurance funds
ICD: International Statistical Classification of Diseases and Related Health Problems
ICC: Intraclass correlation coefficients
PABAK: Prevalence-adjusted bias-adjusted kappa
PIA: Psychiatric outpatient departments
