# Supplementary material for: Agreement between self-reports and statutory health insurance claims data on healthcare utilization in patients with mental disorders
Source: BMC Health Serv Res. 2023 Nov 11;23:1243. doi: 10.1186/s12913-023-10175-6 (PMC10640759; doi:10.1186/s12913-023-10175-6)
Supplement: Supplementary file 1 — Additional file 1: Supplementary Fig. 1. Self-reported utilization of outpatient services and home treatment. Responses were exact number of contacts or frequency. The analysis considered the duration information provided in the questionnaire when the precise number of contacts was stated, otherwise, frequency was taken into consideration. Abbreviations: EP, Established practitioners; PIA, psychiatric outpatient departments. Supplementary Table 1. Variables of primary and secondary data. Supplementary Fig. 2. Venn diagrams illustrate the overlap in the numbers of users (left panels) and non-users (right panels) of (A) inpatient, (B) day-care, (C) outpatient services, and (D) medications in two different data sources. Abbreviations: AR, administrative records; SR, self-reported data. Supplementary Table 2. Proportion of patients by source and concordance between self-reported and administrative data for utilization of health care services and use of medications. All cases with any missing self-report utilization data were excluded. Supplementary Table 3. Differences in healthcare resource use between administrative records and self-reported data for different medical services from the inpatient, day-care, and outpatient settings. All cases with any missing self-report utilization data were excluded. Supplementary Table 4. Differences in healthcare resource use between administrative records and self-reported data for home treatment (n=274). Supplementary Table 5. Differences in medication use between administrative records and self-reported data (n=274). Supplementary Fig. 3. Over- and underreporting on healthcare resource use between administrative records and self-reported data in the inpatient, day-care, and outpatient settings. Discordance was accounted for within a margin of error of ± 1-5 contacts, ± 6-10 contacts, and ± 11 or more contacts. Supplementary Fig. 4. Scatterplots for the correlations between data sources in the inpatient (a), day-care (b) and outpa [file 12913_2023_10175_MOESM1_ESM.docx]

Additional file

#

# Additional information on the data analysis

Use of inpatient and day-care services was assessed by using either information on dates of admission/discharge or information on duration of care in days. When the number of days was stated in the questionnaire, this duration was used in the analysis, otherwise number of days was obtained using dates of admission and discharge informed by the participant. To avoid improbable or inaccurate values, a maximum number of 182 days for inpatient services and 130 days for day-care services was allowed, considering that day-care is performed usually during weekdays.


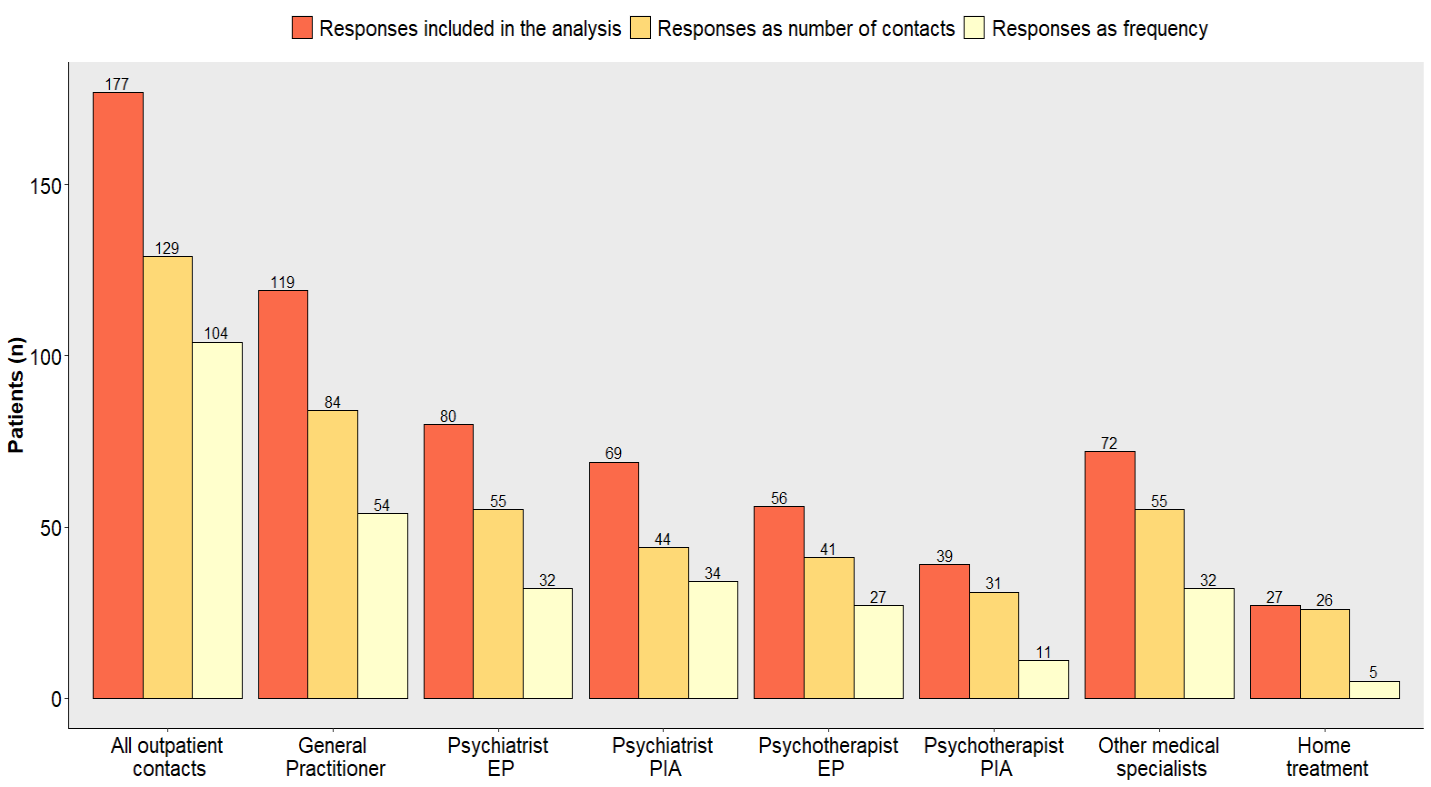
Utilization of outpatient services and home treatment was assessed either as the exact number of contacts or as a frequency (e.g., once a week), then multiplied by the recall period length to estimate the total number of contacts (supplementary Fig. 1). The analysis considered the duration information provided in the questionnaire when the precise number of contacts was stated; otherwise, frequency was taken into consideration. Nonsense open-ended responses (e.g., “???”) or responses that lacked interpretability such as those containing non-substantive information (e.g., “when necessary”) were excluded. To calculate the mean number of contacts based on self-reported frequency of events, we adopted the following: daily was equivalent to 130 contacts (without weekends), twice a week to 52, once a week to 26, every 2 weeks to 13, once a month to 6.

**Supplementary Fig. 1** Self-reported utilization of outpatient services and home treatment. Responses were exact number of contacts or frequency. The analysis considered the duration information provided in the questionnaire when the precise number of contacts was stated, otherwise, frequency was taken into consideration. Abbreviations: EP, Established practitioners; PIA, psychiatric outpatient departments.

To evaluate medication use, the Anatomical Therapeutic Chemical code (ATC) was used to indicate whether participants reported taking any medication, psychotropic medications, or non-psychotropic medications. For psychotropic medications, we defined distinct therapeutic subgroups using 4-digit codes (N05A, antipsychotics; N05B, anxiolytics; N05C, hypnotics and sedatives) or 3-digit code (N06, psychoanaleptics). We excluded drugs that could not be identified, because patients did not fill in the correct German central pharmacy number (PZN) or medication names were unclear. Since patients may use prescription drugs at some time after they were provided, a fixed-time window approach was employed. Medication users in claims data were defined as participants who had been dispensed at least one prescription within 3 months before index date (the day the participants completed the questionnaire). For all other analyses, information from secondary data was extracted for the same period as covered by the primary data (6 months).

To display the overlap between administrative data and self-reported data on healthcare utilization, we built Venn diagrams using the VennDiagram R package [1].

# Additional information on the data sources

**Supplementary Table 1.** Variables of primary and secondary data

| **Variable** | **Indicator CSSRI** | **Indicator SHI** | **Equivalence** |
| --- | --- | --- | --- |
| Sociodemographic |  |  |  |
| Age | Year of birth | Year of birth | Yes |
| Sex | M/F | M/F | Yes |
| Service utilization |  |  |  |
| *Inpatient setting* |  |  |  |
| Non-psychiatric Units of General Hospital | Number of days | Date of admission.  Date of discharge | Yes |
| Psychiatry |  |  |  |
| Psychotherapy |  |  |  |
| Psychosomatics Department |  |  |  |
| Addiction and Substance Misuse |  |  |  |
| Rehabilitation-Addiction. substance misuse | Number of days | n.a. | No |
| Forensic Psychiatry |  |  |  |
| Psychiatric Rehabilitation |  |  |  |
| *Day-care Services* |  |  |  |
| Psychiatric day clinic  Non-psychiatric day clinic | Number of days | Date of admission.  Date of discharge | Yes |
| *Outpatient setting* |  |  |  |
| Psychiatrist (Established practitioners) | Number of contacts | EBM codes/  PIA-Basic service keys | Yes |
| Psychiatrist (PIA) |  |  |  |
| Psychotherapist (Established practitioners) |  |  |  |
| Psychotherapist (PIA) |  |  |  |
| Home treatment |  |  |  |
| General Practitioner |  |  |  |
| Other medical specialists |  |  |  |
| *Complementary non-medical services* |  |  |  |
| Occupational therapy | Number of contacts | APN | Yes |
| Vocational therapy | Number of contacts | n.a. | No |
| Sheltered workshops |  |  |  |
| Contact point and advice centers |  |  |  |
| Social Psychiatric Service |  |  |  |
| Self-help groups |  |  |  |
| *Other medical or social services* |  |  |  |
| Caregiver | Number of contacts | n.a. | No |
| Debt advice centers |  |  |  |
| *Pharmaceutical Treatments* |  |  |  |
| Psychotropic drugs | Utilization (yes/no)  Drug names  PZN codes Frequency  Dose | ATC codes  PZN codes | Yes |
| Other drugs |  |  |  |

APN, settlement item number (German term: *Abrechnungspositionsnummer*); ATC, Anatomical Therapeutic Chemical code; EBM, uniform assessment standard (German term: *Einheitlicher Bewertungsmaßstab*); PIA, psychiatric outpatient departments (German term: *Psychiatrische Institutsambulanz*); PZN, German identification number for pharmaceutical products (German term: *Pharmazentralnummer*).

# Supplementary results


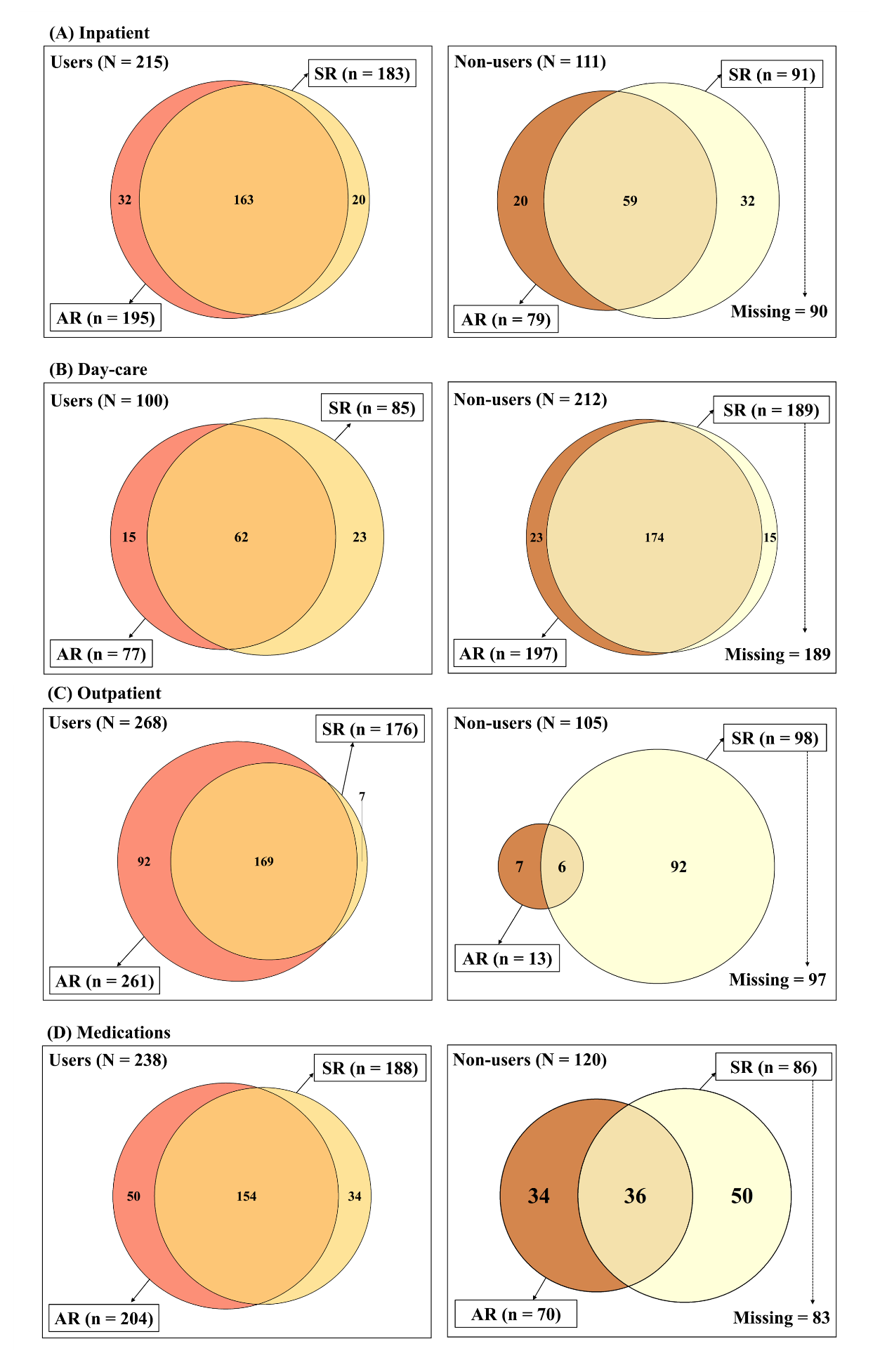


**Supplementary Fig. 2** Venn diagrams illustrate the overlap in the numbers of users (left panels) and non-users (right panels) of (A) inpatient, (B) day-care, (C) outpatient services, and (D) medications in two different data sources. Abbreviations: AR, administrative records; SR, self-reported data.

**Supplementary Table 2.** Proportion of patients by source and concordance between self-reported and administrative data for utilization of health care services and use of medications. All cases with any missing self-report utilization data were excluded.

| Source | Inpatient  (n=184) | Day-care  (n =85) | Outpatient  (n=177) | Medications  (n=191) |
| --- | --- | --- | --- | --- |
| ^(a)^Total utilization - n (%) | 183 (99.5) | 85 (100.0) | 177 (100.0) | 190 (99.5) |
| Utilization in SR - n (%) | 183 (99.5) | 85 (100.0) | 176 (99.4) | 188 (98.4) |
| Utilization in AR - n (%) | 163 (88.6) | 62 (72.9) | 170 (96.0) | 156 (81.7) |
| Utilization in AR and in SR - n (%) | 163 (88.6) | 62 (72.9) | 169 (95.5) | 154 (80.7) |
| Utilization in SR only - n (%) | 20 (10.9) | 23 (27.1) | 7 (4.0) | 34 (17.8) |
| Utilization in AR only - n (%) | 0 (0.0) | 0 (0.0) | 1 (0.6) | 2 (1.1) |
| No utilization in SR and  no utilization in AR- n (%) | 1 (0.5) | 0 (0.0) | 0 (0.0) | 1 (0.5) |
| ^(b)^Agreement - n (%) | 164 (89.1) | 62 (72.9) | 169 (95.5) | 155 (81.2) |
| Kappa [95% CI] | 0.08[-0.07-0.23] | n.a. | -0.01[-0.03-0.01] | 0.02[-0.06-0.11] |
| PABAK | 0.78[0.67-0.86] | n.a. | 0.91[0.83-0.96] | 0.62[0.5-0.73] |
| Sensitivity - % [95% CI] | 4.76[0.2-25.9] | n.a. | n.a. | 2.86[0.1-16.6] |
| Specificity - % [95% CI] | 100[97.1-100] | n.a. | n.a. | 98.72[95-99.8] |

(a)Total number of participants using medical services who were identified by self-reported and/or administrative data; (b)The percentage of agreement indicates concordance between self-reported and administrative data based on the same result (both indicated an event, or both indicated no event). Abbreviations: AR, administrative records; SR, self-reported data; CI, Confidence Interval; kappa, Cohen’s kappa measure of inter-rater agreement; PABAK, Prevalence and Bias Adjusted Kappa; n.a., not applicable.

**Supplementary Table 3.** Differences in healthcare resource use between administrative records and self-reported data for different medical services from the inpatient, day-care, and outpatient settings. All cases with any missing self-report utilization data were excluded.

| Settings | Psychiatric care | Somatic Services | All-cause |
| --- | --- | --- | --- |
| *Inpatient* | **n=174** | **n=18** | **n=180** |
| Number of days SR- Mean (SD) | 50.7 (44.6) | 14.8 (24.2) | 50.51 (44.9) |
| Number of days AR - Mean (SD) | 36.3 (38.2) | 2.0 (7.1) | 38.2 (39.5) |
| Underestimation - n (%) | 85 (48.9) | 5 (27.8) | 92 (51.1) |
| Overestimation - n (%) | 54 (31.0) | 9 (50.0) | 61 (33.9) |
| Total agreement - n (%) | 35 (20.1) | 4 (22.2) | 27 (15.0) |
| ^(a)^Zero in both sources - n (%) | 1 (0.6) | 0 (0.0) | 1 (0.6) |
| ICC [95% CI] | 0.50 [0.39-0.61] | 0.54 [0.13-0.8] | 0.50 [0.38-0.6] |
| ^(b)^*Day-care* | **n=79** | **n=5** | **n=82** |
| Number of days SR- Mean (SD) | 47.1 (37.8) | 74.2 (53.6) | 49.9 (39.0) |
| Number of days AR - Mean (SD) | 10.5 (23.2) | 0 (0.0) | 10.5 (23.2) |
| Underestimation - n (%) | 13 (16.4) | 0 (0.0) | 12 (14.6) |
| Overestimation - n (%) | 54 (68.4) | 5 (100.0) | 58 (70.8) |
| Total agreement - n (%) | 12 (15.2) | 0 (0.0) | 12 (14.6) |
| ^(a)^Zero in both sources - n (%) | 0 (0.0) | 0 (0.0) | 0 (0) |
| ICC [95% CI] | 0.59 [0.34-0.74] | * | 0.47 [0.2-0.66] |
| *Outpatient* | **n=141** | **n=124** | **n=173** |
| Number of contacts SR- Mean (SD) | 8.7 (9.5) | 5.8 (4.9) | 11.3 (10.4) |
| Number of contacts AR - Mean (SD) | 4.6 (10.1) | 7.3 (6.6) | 12.0 (12.1) |
| Underestimation - n (%) | 36 (25.6) | 77 (62.1) | 89 (51.4) |
| Overestimation - n (%) | 89 (63.1) | 33 (26.6) | 72 (41.6) |
| Total agreement - n (%) | 16 (11.3) | 14 (11.3) | 12 (7.0) |
| ^(a)^Zero in both sources - n (%) | 3 (2.1) | 1 (0.8) | 0 (0) |
| ICC [95% CI] | 0.24 [0.08-0.38] | 0.39 [0.21-0.54] | 0.29 [0.14-0.41] |

For calculation purposes, administrative records were treated as the reference for resource utilization. Abbreviations: AR, administrative records; SR, self-reported data; ICC, intraclass correlation coefficient; 95% CI, 95% confidence interval; SD, Standard Deviation. ^(a)^Self-reported data and administrative data indicate no event; ^(b)^Somatic services were not found in administrative data. *Too few cases to analyze.

**Supplementary Table 4.** Differences in healthcare resource use between administrative records and self-reported data for home treatment (n=274)

| Home Treatment | All-cause |
| --- | --- |
| Dichotomous reporting |  |
| Missing SR - n (%) | 247 (90.1) |
| ^(a)^Total utilization - n (%) | 25 (9.1) |
| Utilization in SR - n (%) | 6 (2.2) |
| Utilization in AR - n (%) | 20 (7.3) |
| Utilization in AR and in SR - n (%) | 1 (0.4) |
| Utilization in SR only - n (%) | 5 (1.8) |
| Utilization in AR only - n (%) | 19 (6.9) |
| No utilization in SR, no utilization in AR- n (%) | 249 (90.9) |
| ^(b)^Agreement - n (%) | 250 (91.2) |
| Kappa [95% CI] | 0.04 |
| PABAK | 0.82 |
| Sensitivity - % [95% CI] | [-0.1-0.19] |
| Specificity - % [95% CI] | [0.74-0.88] |
| Quantity reporting |  |
| Missing SR - n (%) | 247 (90.1) |
| Number of days SR- Mean (SD) | 0.99 (8.1) |
| Number of days AR - Mean (SD) | 0.38 (2.2) |
| Underestimation - n (%) | 20 (7.3) |
| Overestimation - n (%) | 5 (1.8) |
| Total agreement - n (%) | 249 (90.9) |
| ^(c)^Zero in both sources - n (%) | 249 (100) |
| ICC [95% CI] | * |

Abbreviations: AR, administrative records; SR, self-reported data; kappa, Cohen’s kappa measure of inter-rater agreement; PABAK, Prevalence and Bias Adjusted Kappa; ICC, intraclass correlation coefficient; 95% CI, 95% confidence interval; SD, Standard Deviation. ^(a)^Total number of participants using medical services who were identified by self-reported and/or administrative data; ^(b)^The percentage of agreement indicates concordance between self-reported and administrative data based on the same result (both indicated an event, or both indicated no event); ^(c)^Number of patients with both responses zero (self-reported data and administrative data indicate no event); *Too few cases to analyze.

**Supplementary Table 5.** Differences in medication use between administrative records and self-reported data (n=274)

| **Drug Class** |  | **SR** | | **^(a)^Utilization in AR (%)** | **Sensitivity**  **(%) [95 CI]** | **Specificity**  **(%) [95 CI]** | **Kappa** | **PABAK** |
| --- | --- | --- | --- | --- | --- | --- | --- | --- |
|  | **AR** | **Yes** | **No** |  |  |  |  |  |
| ***Psychotropic Drugs*** | Yes | 110 | 47 | 55.1 | 70.1  [62.2-77.1] | 65.0  [55.6-73.6] | 0.35 | 0.36 |
|  | No | 41 | 76 |  |  |  |  |  |
| ***Antipsychotics*** | Yes | 53 | 37 | 28.1 | 58.9  [48.0-69.2] | 87.0  [81.2-91.5] | 0.48 | 0.55 |
|  | No | 24 | 160 |  |  |  |  |  |
| ***Anxiolytics*** | Yes | 9 | 7 | 8.4 | 56.2  [29.9-80.2] | 94.6  [91.1-97.0] | 0.42 | 0.85 |
|  | No | 14 | 244 |  |  |  |  |  |
| ***Hypnotics and Sedatives*** | Yes | 7 | 12 | 9.8 | 36.8  [16.3-61.6] | 92.2  [88.2-95.1] | 0.24 | 0.77 |
|  | No | 20 | 235 |  |  |  |  |  |
| ***Psychoanaleptics*** | Yes | 70 | 35 | 38.0 | 66.7  [56.8-75.6] | 79.9  [73.0-85.6] | 0.47 | 0.5 |
|  | No | 34 | 135 |  |  |  |  |  |
| ***Other*** | Yes | 80 | 16 | 55.8 | 83.3  [74.4-90.2] | 59.0  [51.4-66.3] | 0.37 | 0.35 |
|  | No | 73 | 105 |  |  |  |  |  |

The administrative records were used as a reference to calculate sensitivity and specificity. ^(a)^Prevalence of medication accord to administrative records. 95% CI. 95% confidence interval. Abbreviations: AR, administrative records; SR, self-reported data; CI, Confidence Interval; kappa, Cohen’s kappa measure of inter-rater agreement; PABAK, Prevalence and Bias Adjusted Kappa.


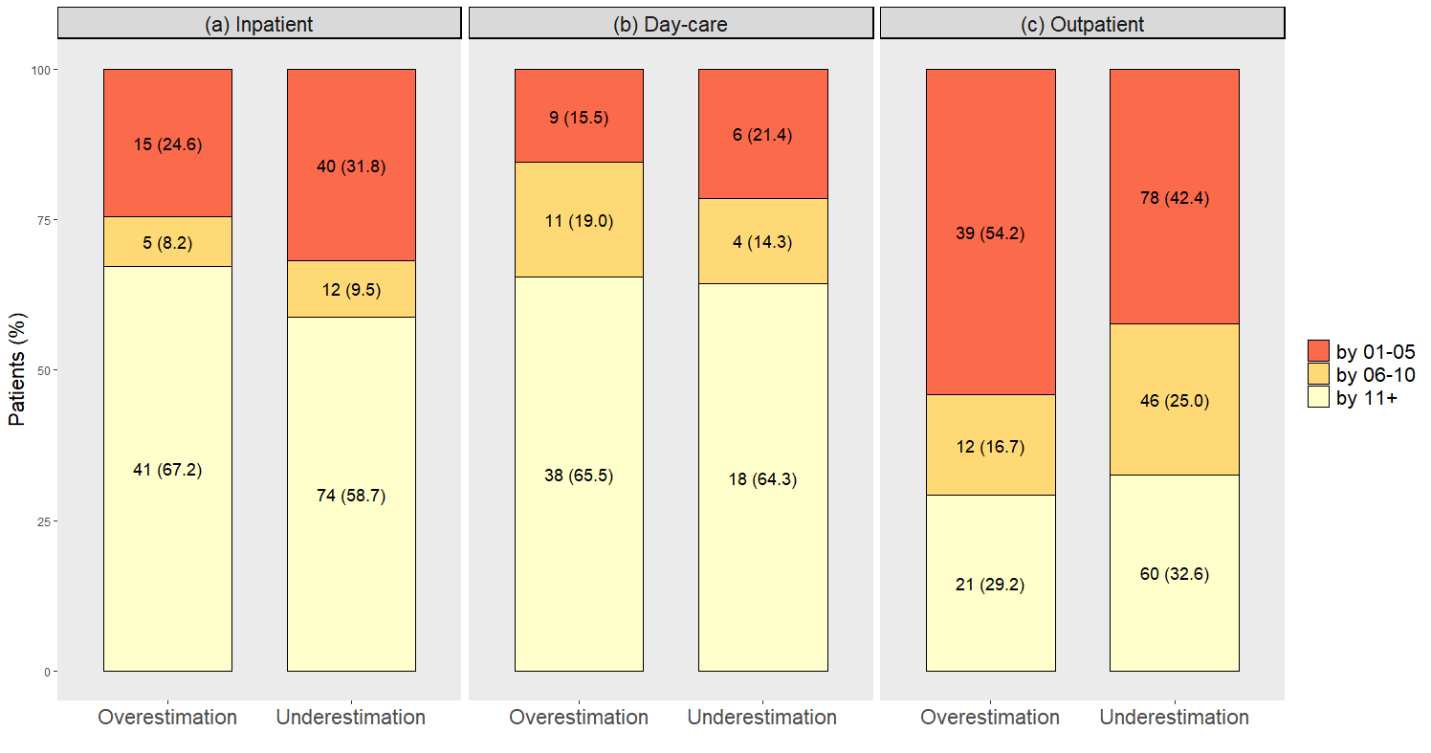


**Supplementary Fig. 3** Over- and underreporting on healthcare resource use between administrative records and self-reported data in the inpatient, day-care, and outpatient settings. Discordance was accounted for within a margin of error of ± 1-5 contacts, ± 6-10 contacts, and ± 11 or more contacts.

**
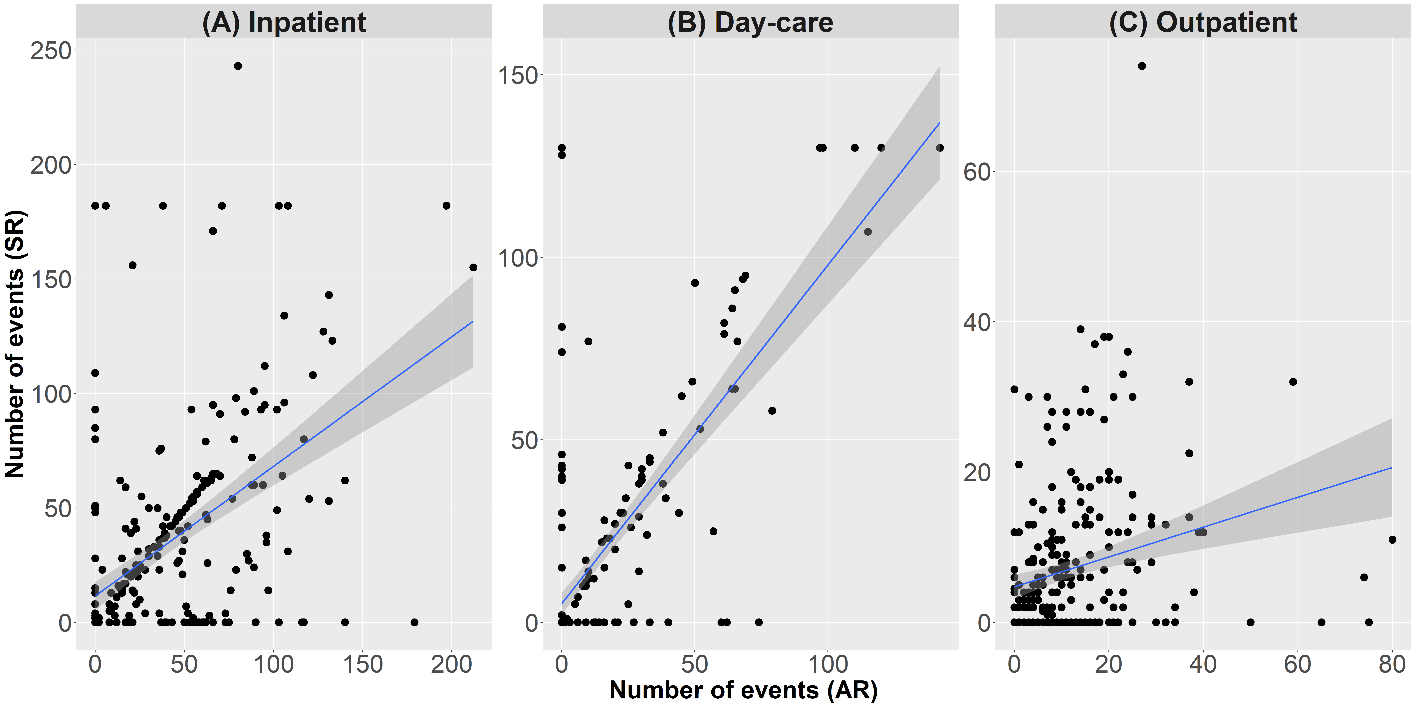
**

**Supplementary Fig. 4** Scatterplots for the correlations between data sources in the inpatient (a), day-care (b) and outpatient (c) settings for the same period.

**Supplementary Table 6.** Correlation coefficients between administrative and self-reported data

| Setting | Correlation - Spearman's rho |
| --- | --- |
| Inpatient (All-cause) | 0.58 (p ≤ 0.001) |
| *(Acute) psychiatry* | 0.51 (p ≤ 0.001) |
| *Psychotherapy* | -0.01 (p=0.83) |
| *Psychosomatics Department* | 0.57 (p ≤ 0.001) |
| *Addiction and Substance Misuse* | 0.10 (p=0.09) |
| Day-care (All-cause) | 0.70 (p ≤ 0.001) |
| Outpatient (All-cause) | 0.31 (p ≤ 0.001) |
| *General Practitioner* | 0.25 (p ≤ 0.001) |
| *Medical specialists - Overall* | 0.40 (p ≤ 0.001) |
| *Established practitioners - Psychiatrist* | 0.34 (p ≤ 0.001) |
| *Established practitioners - Psychotherapist* | 0.45 (p ≤ 0.001) |
| *PIA- Psychiatrist/ Psychotherapist* | 0.41 (p ≤ 0.001) |
| *Other medical specialists* | 0.37 (p ≤ 0.001) |
| Home Treatment | 0.06 (p=0.34) |

**Supplementary Table 7.** Differences in healthcare resource use between administrative records and self-reported data for different medical services from the inpatient setting (n=274)

| Inpatient Setting | Overall |
| --- | --- |
| *(Acute) psychiatry* |  |
| ^(a)^Missing SR - n (%) | 122 (44.5) |
| Utilization in SR - n (%) | 150 (54.7) |
| Utilization in AR - n (%) | 191 (69.7) |
| Number of days SR- Mean (SD) | 28.8 (41.3) |
| Number of days AR - Mean (SD) | 35.2 (36.8) |
| Underestimation - n (%) | 136 (49.6) |
| Overestimation - n (%) | 45 (16.4) |
| Total agreement - n (%) | 93 (33.9) |
| ^(b)^Zero in both sources - n (%) | 67 (72.0) |
| ICC [95% CI] | 0.50 [0.4-0.58] |
| *Psychotherapy* |  |
| ^(a)^Missing SR - n (%) | 270 (98.5) |
| Utilization in SR - n (%) | 4 (1.4) |
| Utilization in AR - n (%) | 3 (1.1) |
| Number of days SR- Mean (SD) | 0.4 (3.2) |
| Number of days AR - Mean (SD) | 0.4 (4.2) |
| Underestimation - n (%) | 3 (1.1) |
| Overestimation - n (%) | 4 (1.5) |
| Total agreement - n (%) | 267 (97.4) |
| ^(b)^Zero in both sources - n (%) | 267 (100) |
| ICC [95% CI] | * |
| *Psychosomatics Department* |  |
| ^(a)^Missing SR - n (%) | 270 (98.5) |
| Utilization in SR - n (%) | 4 (1.4) |
| Utilization in AR - n (%) | 3 (1.1) |
| Number of days SR- Mean (SD) | 1.3 (12.5) |
| Number of days AR - Mean (SD) | 0.4 (4.2) |
| Underestimation - n (%) | 1 (0.4) |
| Overestimation - n (%) | 4 (1.5) |
| Total agreement - n (%) | 269 (98.2) |
| ^(b)^Zero in both sources - n (%) | 269 (100.0) |
| ICC [95% CI] | 0.37 [0.26-0.47] |
| *Addiction and Substance Misuse* |  |
| ^(a)^Missing SR - n (%) | 246 (89.8) |
| Utilization in SR - n (%) | 17 (6.2) |
| Utilization in AR - n (%) | 2 (0.7) |
| Number of days SR- Mean (SD) | 1.8 (6.5) |
| Number of days AR - Mean (SD) | 0.2 (2.6) |
| Underestimation - n (%) | 2 (0.7) |
| Overestimation - n (%) | 27 (9.8) |
| Total agreement - n (%) | 245 (89.4) |
| ^(b)^Zero in both sources - n (%) | 245 (100.0) |
| ICC [95% CI] | * |

Abbreviations: AR, administrative records; SR, self-reported data; ICC, intraclass correlation coefficient; 95% CI, 95% confidence interval; SD, Standard Deviation. ^(a)^In the statistical analyses, answers “zero (0)” and missing in the questionnaire were merged into “no utilization”; ^(b)^Number of patients with both responses zero (self-reported data and administrative data indicate no event); *Too few cases to analyze.

**Supplementary Table 8.** Differences in healthcare resource use between administrative records and self-reported data for different medical services from the outpatient setting (n=274)

| Outpatient Setting | Overall |
| --- | --- |
| *General Practitioner* |  |
| ^(a)^Missing SR - n (%) | 159 (58.0) |
| Utilization in SR - n (%) | 108 (39.4) |
| Utilization in AR - n (%) | 233 (85.0) |
| Number of contacts SR- Mean (SD) | 1.8 (3.2) |
| Number of contacts AR - Mean (SD) | 4.5 (4.4) |
| Underestimation - n (%) | 188 (68.6) |
| Overestimation - n (%) | 33 (12.0) |
| Total agreement - n (%) | 53 (19.3) |
| ^(b)^Zero in both sources - n (%) | 32 (60.4) |
| ICC [95% CI] | 0.24 [0.07-0.38] |
| *Medical specialists - Overall* |  |
| ^(a)^Missing SR - n (%) | 117 (42.7) |
| Utilization in SR - n (%) | 154 (56.2) |
| Utilization in AR - n (%) | 207 (75.5) |
| Number of contacts SR- Mean (SD) | 5.4 (8.7) |
| Number of contacts AR - Mean (SD) | 4.6 (5.5) |
| Underestimation - n (%) | 123 (44.9) |
| Overestimation - n (%) | 93 (33.9) |
| Total agreement - n (%) | 58 (21.2) |
| ^(b)^Zero in both sources - n (%) | 45 (77.6) |
| ICC [95% CI] | 0.30 [0.19-0.4] |
| *Established practitioners -* *Psychiatrist* |  |
| ^(a)^Missing SR - n (%) | 197 (71.9) |
| Utilization in SR - n (%) | 65 (23.7) |
| Utilization in AR - n (%) | 41 (15.0) |
| Number of contacts SR- Mean (SD) | 1.0 (2.5) |
| Number of contacts AR - Mean (SD) | 0.6 (1.7) |
| Underestimation - n (%) | 29 (10.6) |
| Overestimation - n (%) | 49 (17.9) |
| Total agreement - n (%) | 196 (71.5) |
| ^(b)^Zero in both sources - n (%) | 192 (98.0) |
| ICC [95% CI] | 0.30 [0.12-0.34] |
| *Established practitioners -* *Psychotherapist* |  |
| ^(a)^Missing SR - n (%) | 221 (80.7) |
| Utilization in SR - n (%) | 36 (13.1) |
| Utilization in AR - n (%) | 59 (21.5) |
| Number of contacts SR- Mean (SD) | 1.5 (5.2) |
| Number of contacts AR - Mean (SD) | 1.1 (2.7) |
| Underestimation - n (%) | 44 (16.1) |
| Overestimation - n (%) | 24 (8.8) |
| Total agreement - n (%) | 206 (75.2) |
| ^(b)^Zero in both sources - n (%) | 204 (99.0) |
| ICC [95% CI] | 0.24 [0.13-0.35] |
| *PIA- Psychiatrist/ Psychotherapist* |  |
| ^(a)^Missing SR - n (%) | 198 (72.3) |
| Utilization in SR - n (%) | 61 (22.3) |
| Utilization in AR - n (%) | 112 (40.9) |
| Number of contacts SR- Mean (SD) | 2.0 (5.7) |
| Number of contacts AR - Mean (SD) | 3.0 (9.3) |
| Underestimation - n (%) | 76 (27.7) |
| Overestimation - n (%) | 45 (16.4) |
| Total agreement - n (%) | 153 (55.8) |
| ^(b)^Zero in both sources - n (%) | 146 (95.4) |
| ICC [95% CI] | 0.22 [0.1-0.33] |
| *Other medical specialists* |  |
| ^(a)^Missing SR - n (%) | 211 (77.0) |
| Utilization in SR - n (%) | 63 (23.0) |
| Utilization in AR - n (%) | 180 (65.7) |
| Number of contacts SR- Mean (SD) | 0.9 (2.2) |
| Number of contacts AR - Mean (SD) | 2.8 (3.8) |
| Underestimation - n (%) | 150 (54.7) |
| Overestimation - n (%) | 19 (6.9) |
| Total agreement - n (%) | 105 (38.3) |
| ^(b)^Zero in both sources - n (%) | 92 (87.6) |
| ICC [95% CI] | 0.23 [0.08-0.36] |

Abbreviations: AR, administrative records; SR, self-reported data; ICC, intraclass correlation coefficient; PIA, psychiatric outpatient departments; 95% CI, 95% confidence interval; SD, Standard Deviation. ^(a)^In the statistical analyses, answers “zero (0)” and missing in the questionnaire were merged into “no utilization”; ^(b)^Number of patients with both responses zero (self-reported data and administrative data indicate no event).

# References

[1] Chen H, Boutros PC. VennDiagram: a package for the generation of highly-customizable Venn and Euler diagrams in R. BMC Bioinformatics. 2011;12(1):35. doi:10.1186/1471-2105-12-35
